# Clinical Verifications

**Published:** 1887-09

**Authors:** G. W. Sherbino

**Affiliations:** Abilene, Texas


### CLINICAL VERIFICATIONS.

G. W. Sherbino, M. D., Abilene, Texas.

*Lac. Can.*$^{mm}$ (*Swan's*).—*Headache* (pain changing from one supraorbital region to the other).

Dull pain that had lasted for several days—"To-day on one side, to-morrow on the other." (Erratic, Puls., Sulph.)

One dose at night; pain got better; came back next day. One more dose relieved permanently.

*Bryonia Alb.*$^{5cm}$, *B. & T.*—Patient, recovering from dengue, was taken with a pain in the region of the heart, pain extending like a flash back to the right axilla. At the same time a pain in the right illiac, which she had to grasp with her hand and hold tight. Exacerbated by any motion, "laughing" or "talking."

One dose "cured."

*Tarantula*$^{cm}$.—Mrs. ——, æt. forty, has been treated for heart disease by the old school, and when they fail, "which they usually do," the patient tries to find a disciple of Hahnemann. She is taken with extremely nervous spells, with a pain in the left ovary, and pain then seems to affect her heart and she becomes unconscious; seems to stop breathing. Use artificial respiration, and in a short time she will draw a long breath, look around, and begin to breathe.

As the spell comes on again she begins to breathe short and call for more air; pulls her dress away from her heart; constantly pulling her dress; gasping for air; all of a sudden she becomes unconscious, and the breathing seems to stop. The pulse intermits, weak and slow. Many times she is reported dying. Called at the office one day, and I obtained the following symptoms:

Great oppression of the chest.

Panting respiration. Palpitation of the heart. Trembling and thumping of the heart as from fright.

Suffocation. Constant desire for air. Heart suddenly ceases to beat. Sensation as if the heart was squeezed or compressed. (Cact., Iod., Diosc., Lilium tig.)

She said she could not feel her heart at all, any more than as if she had none. Tarantula cured, and has never had another attack now for more than a year.

